# Patient-important outcomes reported in randomized controlled trials of pharmacologic treatments for COVID-19: a protocol of a META-epidemiological study

**DOI:** 10.1186/s13643-021-01838-8

**Published:** 2021-11-01

**Authors:** Mario A. Jimenez-Mora, Andrea Ramírez Varela, Jose F. Meneses-Echavez, Julia Bidonde, Adriana Angarita-Fonseca, Reed A. C. Siemieniuk, Dena Zeraatkar, Jessica J. Bartoszko, Romina Brignardello-Petersen, Kimia Honarmand, Bram Rochwerg, Gordon Guyatt, Juan José Yepes-Nuñez

**Affiliations:** 1 School of Medicine, Universidad de los Andes, Bogotá, 111711 Colombia; 2 Division for Health Services, Norwegian Institute of Public Health, Oslo, Norway; 3 School of Rehabilitation Science, Univ of Saskatchewan, Saskatoon, SK Canada; 4 Université de Montréal, Montréal, Quebec, Canada; 5 Universidad de Santander, Bucaramanga, Santander Colombia; 6 Department of Health Research Methods, Evidence, and Impact, McMaster University, Hamilton, ON Canada; 7 Division of Critical Care, Department of Medicine, Western University, London, ON Canada; 8 Department of Medicine, McMaster University, Hamilton, ON Canada; 9 Pulmonology Service, Internal Medicine Section, Fundación Santa Fe de Bogotá University Hospital, Bogotá, Colombia

**Keywords:** Patient-important outcome, Reporting, Treatment, Prevention, Coronavirus, Covid-19

## Abstract

**Background:**

The coronavirus disease 19 (covid-19) pandemic has underscored the need to expedite clinical research, which may lead investigators to shift away from measuring patient-important outcomes (PIO), limiting research applicability. We aim to investigate if randomized controlled trials (RCTs) of covid-19 pharmacological therapies include PIOs.

**Methods:**

We will perform a meta-epidemiological study of RCTs that included people at risk for, or with suspected, probable, or confirmed covid-19, examining any pharmacological treatment or blood product aimed at prophylaxis or treatment. We will obtain data from all RCTs identified in a living network metanalysis (NMA). The main data sources are the living WHO covid-19 database up to 1 March 2021 and six additional Chinese databases up to 20 February 2021. Two reviewers independently will review each citation, full-text article, and abstract data. To categorize the outcomes according to their importance to patients, we will adapt a previously defined hierarchy: a) mortality, b) quality of life/ functional status/symptoms, c) morbidity, and d) surrogate outcomes. Outcomes within the category a) and b) will be considered critically important to patients, and outcomes within the category c) will be regarded as important. We will use descriptive statistics to assess the proportion of studies that report each category of outcomes. We will perform univariable and multivariable analysis to explore associations between trial characteristics and the likelihood of reporting PIOs.

**Discussion:**

The findings from this meta-epidemiological study will help health care professionals and researchers understand if the current covid-19 trials are effectively assessing and reporting the outcomes that are important to patients. If a deficiency in capturing PIOs is identified, this information may help inform the development of future RCTs in covid-19.

**Systematic review registrations:**

Open Science Framework registration: osf.io/6xgjz.

**Supplementary Information:**

The online version contains supplementary material available at 10.1186/s13643-021-01838-8.

## Background

Healthcare systems worldwide have called for the increased involvement of lay people, which has led public and patients to become more active in their own health and health care decisions. Patient-important outcomes (PIO) are gaining wide acceptance in most fields of clinical research with clinicians and researchers advocating for “the patient at the center” of medical decision making [1].

This trend has provoked a shift when recommending interventions. In the past, the magnitude of the effect as “clinically relevant” was considered as the most relevant factor; judgments have replaced this as to whether effects are “patient-important” [1]. The notion of “patient-important” sheds light on the individual clinical encounter and the importance of patients’ values and preferences. In clinical research, a PIO has been previously defined as: “a characteristic or variable that reflect how a patient feels, functions or survives” [2, 3].

This change in clinical decision-making paradigm represent a shift from a parental model of care in which the clinician decides the relevant outcomes to a patient-centered approach involving patients’ values and preferences [1]. This shift becomes especially important when one considers that, despite compelling physiological rationale, interventions’ that impact on surrogate outcomes such as laboratory results often fail to achieve patient-important benefit [4].

Thus, including PIOs become of essential importance when assessing the benefits and harms of a new therapy. However, despite this potential, PIOs are often not the focus of clinical trialists. Because a focus on surrogates results in trials are faster, cheaper, and require fewer patients, so many trialists focus on endpoints that are not in themselves important [5]. The Covid-NMA project [6] is an example where researchers had reminded trialist their outcomes had to be consistent with the core outcome sets developed by the COMET (Core Outcome Measures in Effectiveness Trials) initiative to enable their trial to be incorporated into Covid NMA meta-analysis. Health care providers and administrators perceive time to collect and input data as the greatest barriers to PIO implementation [7]. However, given the repeated failure of intervention effects on surrogate outcomes to align with effects important to patients, the result may be implementation of useless or even harmful interventions [8].

In the coronavirus disease 19 (covid-19) pandemic, “thousands of trials have been planned to identify treatments and preventive interventions for coronavirus disease 2019 (COVID-19)” [9]. Researchers are rushing to test pharmacological interventions that may decrease the personal and social burden of the disease as well as producing associated guideline recommendations [10]. Current literature, however, points to some methodological shortcuts trialist have taken such as low transparency, high heterogeneity, and suboptimal statistical methods [11]. Trials reportedly have small sample size are single center, and present with redundancy in research questions [9]. The need to obtain expedient answers may lead investigators to shift away from PIOs, which can lead to unresolved questions regarding the benefits and harms of the treatments. In an effort to standardize outcome measures in covd-19 studies, the World Health Organization has developed a minimal set of common outcome measures for ongoing and future covid-19 studies [12]. With hundreds of current and future covid-19 randomized controlled trials (RCTs) ongoing or planned, it is essential to understand the outcomes being evaluated.

## Methods

The protocol for this meta-epidemiological study is registered in Open Science Framework (OSF) (osf.io/6xgjz) and the study will be conducted and reported in line with international standards [13, 14]. For the reporting of the present protocol, we used to the Preferred Reporting Items for Systematic Review and Meta-Analysis Protocols (PRISMA-P) [15] (see Additional File 1). This project is associated to a recently published living systematic review and network metanalysis (NMA) that compared the effects of treatments for covid-19 [16].

### Data source and eligibility criteria

We will obtain data from all RCTs identified in a recent living systematic review and NMA [16] that utilized a comprehensive multilingual source of global covid-19 literature, up to 1 March 2021 and six additional Chinese databases up to 20 February 2021. Studies identified as of 12 February 2021 were included in this analysis. The WHO literature search strategy [16] is provided in Additional file 2.

RCTs will be included if include people at risk for, or with suspected, probable, or confirmed covid-19, or include any pharmacological treatment or blood products intervention aimed either at prophylaxis or treatment vs placebo, standard care, an alternative intervention and/or no intervention, or their publication status is stated as peer reviewed, in press, or preprint or is written in any language.

We will apply no restrictions based on illness severity or setting. Additional file 3 presents example of pharmacological treatment for covid 19. Studies comparing any of the interventions listed in Additional file X with each other or placebo will be eligible for inclusion. We will exclude randomized trials evaluating vaccination, blood products and antibody-based antiviral therapies (such as virus-specific monoclonal antibodies), nutrition, traditional Chinese herbal or alternative medicines that include more than one molecule or a molecule without specific molecular weighted dosing, and non-drug supportive care interventions.

### Data extraction and coding

We will use a standardized data extraction form to collect the data from included studies. Pairs of reviewers will extract data and classify the outcomes independently and in duplicate. Disagreements between reviewers will be resolved through discussion and consensus. If necessary, a third reviewer will be consulted.

The following details will be extracted:

- Type of publication (peer-reviewed vs. preprint)
- Patient characteristics (age, gender, disease severity, and comorbidities)
- Covid-19 diagnosis method (nucleic tests vs. clinical criteria)
- Publication characteristics (country, type of funding, trial phase, sample size, design, whether the study protocol had been previously published
- Interventions’ characteristics (doses, routes and duration of administration)
- Outcomes (standard mean or standard mean difference for continuous outcomes, and odds ratio or relative risk for dichotomous outcomes).

We will extract information for all outcomes (primary and other), along with their corresponding definition. We will categorize the outcomes according to their importance to patients adopting a previously established hierarchy [17–19], which was adjusted to include, but not limited to, relevant outcomes in COVID-19, as follows:

**Table:** 

a) Mortality	• all-cause mortality • disease-specific mortality
b) Quality of life / Functional status / Symptoms	• mental health • physical health • prevalence of fever • clearance time of fever • prevalence of dyspnea, • clearance recovery time score of clinical symptoms • pneumonia severity index
c) Morbidity	• respiratory support (e.g., respiratory failure occurred, need for respiratory support, mechanical ventilation required, duration of mechanical ventilation, frequency of requirement for mechanical ventilation, ventilator free days, need for non-invasive mechanical ventilation, including noninvasive ventilation (NIV) and high flow nasal cannula (HFNC), prevalence of mechanical ventilation) • cardiovascular major morbid events (e.g. stroke, myocardial infarction) • other major morbid events (e.g., intensive care unit (ICU) admission, shock occurrence, prevalence and time of progressing to severe or critical types, renal replacement therapy, vasopressor support) • incidence/prevalence of severe acute respiratory distress syndrome (ARDS) coronavirus 2 disease (e.g., recovery prevalence) and other chronic diseases (e.g., COPD exacerbation, new onset of diabetes) • hospitalization, medical and surgical procedures (e.g., length of hospital stay, time to achieve a normal respiratory rate, rate of survival,) • organ failure (in addition to pulmonary) • adverse events or side effects (any)
d) Surrogate outcomes	• viral load, virus antibody level, oxygen saturation, arterial blood-gas analysis, lymphocyte count, PaO2/FiO2, C-reactive protein (CRP) level, time for CRP return to baseline, time to negative conversion of severe acute respiratory syndrome coronavirus 2, lesions progression within 24–48 h in pulmonary imaging

Outcomes within the category a) and b) will be considered critically important to patients, and outcomes within the category c) will be regarded as important [17]. Outcomes within category d) will be regarded as not patient-important. We will identify the “most PIO” of each study as the outcome that pertains to the highest level of the hierarchy [19]. For the primary outcome (as reported by the authors) and the most PIO, we will determine if it is one of benefit or harm and will extract the relative and the absolute effect estimates used to report the outcome results.

#### Risk of Bias

Data on the risk of bias assessment will be obtained from the original living systematic review and NMA [16]. In the living systematic review and NMA, for each eligible trial, reviewers used a revision of the Cochrane tool for assessing risk of bias in randomized trials (RoB 2.0) to rate trials as either at (i) low risk of bias, (ii) some concerns—probably low risk of bias, (iii) some concerns—probably high risk of bias, or (iv) high risk of bias, across the following domains: bias arising from the randomization process; bias owing to departures from the intended intervention; bias from missing outcome data; bias in measurement of the outcome; bias in selection of the reported results, including deviations from the registered protocol; bias due to competing risks; and bias arising from early termination for benefit. We rated trials at high risk of bias overall if one or more domains were rated as probably high risk of bias or as high risk of bias and as low risk of bias if all domains were rated as probably low risk of bias or low risk of bias. For ivermectin, the linked guideline panel also requested a review of the study protocols to check that trial registration occurred prior to patient recruitment. Reviewers resolved discrepancies by discussion and, when not possible, with adjudication by a third reviewer [13].

### Data synthesis

We will use descriptive statistics to summarize the study characteristics. We will describe continuous variables with median and interquartile range (IQR), and categorical variables are with frequencies and percentages. We will use frequencies and percentages to describe the proportion of studies that report: each individual outcome in each study, each outcome category (a to d), a PIO (category a or b or c) as the primary outcome of the study, and the most PIO as the primary outcome of the study. We will summarize this information in tabular formats.

We will apply a univariate analysis to explored associations between trial characteristics and likelihood of reporting PIO, using Chi-square test or Fisher’s test for categorical variables and Student’s test or Wilcoxon’s test for continuous variables. We will conduct a multivariate analysis to test predictors, using logistic regression. For this analysis the dependent variable will be the PIO (yes/ no) as the dependent variable in the case a trial included PIO as the primary outcome. We plan to conduct more than one multivariable analysis because the dependent variable can be different according to the most patient important outcome (category a or b or c). The independent predictors will include: 1) type of funding (industry supported vs non-industry supported); 2) number of patients (every additional 100 patients); 3) severity of disease (mild/moderate or mixed vs severe, in not prophylactic RCTs); 4) follow-up duration (for every additional 30 days); 5) type of publication (regular vs. preprint); 6) risk of bias (high vs low); 7) prophylaxis trials vs treatment trials; and 8) pharmacological interventions vs blood products. The rationale for choosing these predictors is 1) PIO reporting may be more frequent in non-industry supported studies; 2) smaller trials may be less likely to report PIO because they are not powered for these outcomes; 3) trials enrolling patients with severe disease, may be more likely to report PIO; 4) trials with shorter follow- up duration may be less likely to report PIO because mortality and morbidity events possibly require longer time to occur; 5) publication in an indexed journal after peer review are expected to report more PIO; 6) studies that report PIO may display an overall lower risk of bias; 7) prophylaxis trials may be less likely to report PIO; and 8) pharmacological intervention trials may be more likely to report PIO. We will calculate odds ratios (ORs) and their 95% confidence intervals (CI) to determine the strength of association between predictors and outcome.

We will classify the studies as high or low risk of bias using the same threshold reported by Siemieniuk et al. [16]. A study will be classified with an overall high risk of bias if one or more domains were rated as some concerns- probably high risk of bias- or as high risk of bias; and as overall low risk of bias if all domains were rated as some concerns-probably low risk of bias- or low risk of bias. This systematic review is nested in a previously published NMA, which will be updated. According to Peduzzi et al. [20]., the minimum required sample size should be based on the rule of 10 event per independent variable. Since in the published NMA, 39 out of 41 trials evaluated PIOs and the planned number of predictors is 8, we will include all trials that meet the inclusion and exclusion criteria. We estimated that we need at least 80 trials that measured PIO. However, we need to add 5% (*n* = 4) trials that will not evaluate a PIO and 20% (*n* = 16) that will not evaluate the 8 predictors at the same time. This meant that we need to include 100 trials. Considering that trials are usually unpowered to detect predetermined significant differences in their primary outcomes, we considered primary outcomes in our main analysis and conducted sensitivity analysis using both primary and secondary outcomes. We will conduct the statistical analysis using Stata 17 (StataCorp. 2021. Stata Statistical Software: Release 17. College Station, TX: StataCorp LLC).

## Discussion

To our knowledge, this protocol describes the first meta-epidemiological study that specifically examines the frequency of PIOs reporting in RCTs in covid-19. Previous studies have described the frequency of PIOs in systematic reviews that included RCTs in diabetes [5] and critically ill patients [21]. Umbrella reviews have also explored the frequency of PIOs included in systematic reviews of therapeutic interventions for different conditions [22], in the full texts and abstracts of Cochrane and non-Cochrane reviews [17], and in a systematic review that explored whether the outcomes reported in the summary of finding tables (SoF) of Cochrane systematic reviews included PIOs [23].

This meta-epidemiological study will describe the proportion of studies that reported individual outcomes and their categories, or PIO as the primary outcome of the study, and the most PIO as the primary outcome of the study. Our target users are health care professionals, as well as researchers, decision makers and other stakeholders.

Limitations to the meta-epidemiological study may include the diversity of outcomes from different clinical trials that may limit our capacity to classify these outcomes in our proposed category. Quantity does not equal quality; the rush to register and publish trials during covid 19 pandemic has, from a methodological point of view, being challenging. Information on protocols of individual studies differs from registry to registry. Furthermore, study designs, criteria for stratification of patients and choice of outcomes are quite heterogeneous. All this makes data sharing and secondary analysis such as this study difficult.

We will face some operational issues in our study. We will include studies reported in English and Chinese and trials that included pharmacological agents for treating covid-19. We expect to include broader trials covering other interventions for preventing and treating covid-19 and studies published in different languages in future analyses. The findings from this meta-epidemiological study will help health care professionals and researchers understand if the current COVID-19 trials are effectively assessing and reporting the outcomes that are relevant to patients. This study will also identify gaps in outcomes reporting which might be useful for further research and priority setting and it will support decision making and the planning of future clinical trials.

We plan to present our results at national and international meetings. we will write up the most important findings of the review for a scientific journal targeting specific audience groups (e.g., doctors) and sub-groups (e.g., patients) the team considers need the review the most. We will distribute the results among organizations known to the authors working in the area like the Society for Critical Care. We will do so in the form of plain language summaries, Tweets, blogs, Instagram and Facebook posts.

## Supplementary Information

**Additional file 1.**

**Additional file 2.**

**Additional file 3.**

## Data Availability

The datasets created and analyzed during this survey will be available from the corresponding author upon reasonable request.

## Abbreviations

ARDS: Acute respiratory distress syndrome
Covid-19: Coronavirus disease 2019
COPD: Chronic obstructive pulmonary disease
CRP: C-reactive protein
ICU: Intensive care unit
NMA: Network metanalysis
NIV: Noninvasive ventilation
HFNC: High flow nasal cannula
PaO2/FiO2: Ratio of arterial oxygen partial pressure to fractional inspired oxygen
PIO: Patient-important outcome
RCT: Randomized clinical trials
