# Supplementary material for: Patient-important outcomes reported in randomized controlled trials of pharmacologic treatments for COVID-19: a protocol of a META-epidemiological study
Source: Syst Rev. 2021 Nov 1;10:289. doi: 10.1186/s13643-021-01838-8 (PMC8559914; doi:10.1186/s13643-021-01838-8)
Supplement: Supplementary file 2 — Additional file 2. [file 13643_2021_1838_MOESM2_ESM.docx]

## Additional File 2. Search strategies.

| **Database** | **Daily Search Strategy** |
| --- | --- |
| Medline (Ovid) 1946- | (coronavir* OR corona virus* OR corona pandemic* OR betacoronavir* OR covid19 OR covid OR nCoV OR novel CoV OR CoV 2 OR CoV2 OR sarscov2 OR sars2 OR 2019nCoV OR wuhan virus*).mp. OR (sars AND cov).mp. OR ((wuhan OR hubei OR huanan) AND (severe acute respiratory OR pneumonia*) AND outbreak*).mp. OR Coronavirus Infections/ OR Coronavirus/ OR betacoronavirus/  Limits: 2020- |
| CAB  Abstracts (Ovid) 1910- | (coronavir* OR corona virus* OR corona pandemic* OR betacoronavir* OR covid19 OR covid OR nCoV OR novel CoV OR CoV 2 OR CoV2 OR sarscov2 OR sars2 OR 2019nCoV OR wuhan virus*).mp. OR (sars AND cov).mp. OR ((wuhan OR hubei OR huanan) AND (severe acute respiratory OR pneumonia*) AND outbreak*).mp. OR exp Betacoronavirus/ |
| Global Health (Ovid) 1910- | (coronavir* OR corona virus* OR corona pandemic* OR betacoronavir* OR covid19 OR covid OR nCoV OR novel CoV OR CoV 2 OR CoV2 OR sarscov2 OR sars2 OR 2019nCoV OR wuhan virus*).mp. OR (sars AND cov).mp. OR ((wuhan OR hubei OR huanan) AND (severe acute respiratory OR pneumonia*) AND outbreak*).mp. OR exp Betacoronavirus/ |
| PsycInfo (Ovid) 1806- | (coronavir* OR corona virus* OR corona pandemic* OR betacoronavir* OR covid19 OR covid OR nCoV OR novel CoV OR CoV 2 OR CoV2 OR sarscov2 OR sars2 OR 2019nCoV OR wuhan virus*).mp. OR (sars AND cov).mp. OR ((wuhan OR hubei OR huanan) AND (severe acute respiratory OR pneumonia*) AND outbreak*).mp.  Limits: 2020- |
| Scopus 1960- | TITLE-ABS-KEY ( coronavir* OR "corona virus" OR "corona pandemic" OR betacoronavir* OR covid19 OR covid OR ncov OR "CoV 2" OR cov2 OR sarscov2 OR sars2 OR 2019ncov OR "novel CoV" OR "wuhan virus" ) OR TITLE-ABS-KEY(sars AND cov) OR ( TITLE-ABS-KEY ( wuhan OR hubei OR huanan ) AND TITLE-ABS-KEY ( "severe acute respiratory" OR pneumonia* ) AND TITLE-ABS-KEY ( outbreak* ) ) AND ( LIMIT-TO ( PUBYEAR , 2021 ) OR LIMIT-TO ( PUBYEAR , 2020 ) ) |
| Academic Search Complete (Ebsco) | TI,AB,SU( ( coronavir* OR "corona virus" OR "corona pandemic" OR betacoronavir* OR covid19 OR covid OR ncov OR "CoV 2" OR cov2 OR sarscov2 OR sars2 OR 2019ncov OR "novel CoV" OR "wuhan virus" ) OR (sars AND cov) OR ( ( wuhan OR hubei OR huanan ) AND ( "severe acute respiratory" OR pneumonia* ) AND ( outbreak* ) ) ) |
