# Supplementary material for: Patient-important outcomes reported in randomized controlled trials of pharmacologic treatments for COVID-19: a protocol of a META-epidemiological study
Source: Syst Rev. 2021 Nov 1;10:289. doi: 10.1186/s13643-021-01838-8 (PMC8559914; doi:10.1186/s13643-021-01838-8)
Supplement: Supplementary file 3 — Additional file 3. [file 13643_2021_1838_MOESM3_ESM.docx]

**Additional File 3. Eligibility criteria**

We will include RCTs in which persons exposed to COVID-19 or with suspected, probable, or

confirmed COVID-19 are treated with pharmacologic or blood products aimed at prophylaxis or

178 treatment. We will include trials in which researchers compare any intervention against another or against no intervention, placebo, or standard of care, and report any outcome. We will include trials that report results regardless of publication status (peer-reviewed, in press, or pre-print, but not news reports alone) or language. There will be no restrictions on acuity of disease, nor setting.

We will include trials of pharmaceuticals, blood products, vitamins, minerals and, if the drug is

one specific molecule, Chinese medicines. We will exclude quasi-randomized studies and

randomized trials evaluating external organ support, plasma exchange, oxygen delivery,

ventilation strategies, vaccination, nutrition, traditional Chinese herbal medicines (that typically

include more than one molecule or a molecule without specific molecular weighted dosing),

exercise/rehabilitation, psychological and educational interventions, personal protective

equipment, or any other non-drug supportive care interventions.

**List of interventions included in the review**

Standard care

Alpha lipoic acid

Baloxavir marboxil

Chloroquine

Glucocorticoids

Diammonium glycyrrhizinate

Favipiravir

Hydroxychloroquine

Interferon beta-1a

Lopinavir-ritonavir

Novaferon

Novaferon, lopinavir-ritonavir

Remdesivir

Ribavirin

Ribavirin, interferon beta-1b

Ribavirin, lopinavir-ritonavir

Ruxolitinib

Umifenovir
